# Diagnostic windows in non-neoplastic diseases: a systematic review

**DOI:** 10.3399/BJGP.2023.0044

**Published:** 2023-06-13

**Authors:** Emma Whitfield, Becky White, Spiros Denaxas, Georgios Lyratzopoulos

**Affiliations:** ECHO (Epidemiology of Cancer Healthcare & Outcomes), Department of Behavioural Science and Health, Institute of Epidemiology and Health Care, University College London (UCL), London, and Institute of Health Informatics, UCL, London.; ECHO (Epidemiology of Cancer Healthcare & Outcomes), Department of Behavioural Science and Health, Institute of Epidemiology and Health Care, University College London (UCL), London.; Institute of Health Informatics, UCL, London; associate director, British Heart Foundation Data Science Centre, London; Health Data Research UK, London, and UCL Hospitals Biomedical Research Centre, London.; ECHO (Epidemiology of Cancer Healthcare & Outcomes), Department of Behavioural Science and Health, Institute of Epidemiology and Health Care, University College London (UCL), London.

**Keywords:** diagnosis, electronic health records, primary health care

## Abstract

**Background:**

Investigating changes in prediagnostic healthcare utilisation can help identify how much earlier conditions could be diagnosed. Such ‘diagnostic windows’ are established for cancer but remain relatively unexplored for non-neoplastic conditions.

**Aim:**

To extract evidence on the presence and length of diagnostic windows for non-neoplastic conditions.

**Design and setting:**

A systematic review of studies of prediagnostic healthcare utilisation was carried out.

**Method:**

A search strategy was developed to identify relevant studies from PubMed and Connected Papers. Data were extracted on prediagnostic healthcare use, and evidence of diagnostic window presence and length was assessed.

**Results:**

Of 4340 studies screened, 27 were included, covering 17 non-neoplastic conditions, including both chronic (for example, Parkinson’s disease) and acute conditions (for example, stroke). Prediagnostic healthcare events included primary care encounters and presentations with relevant symptoms. For 10 conditions, sufficient evidence to determine diagnostic window presence and length was available, ranging from 28 days (herpes simplex encephalitis) to 9 years (ulcerative colitis). For the remaining conditions, diagnostic windows were likely to be present, but insufficient study duration was often a barrier to robustly determining their length, meaning that diagnostic window length may exceed 10 years for coeliac disease, for example.

**Conclusion:**

Evidence of changing healthcare use before diagnosis exists for many non-neoplastic conditions, establishing that early diagnosis is possible, in principle. In particular, some conditions may be detectable many years earlier than they are currently diagnosed. Further research is required to accurately estimate diagnostic windows and to determine how much earlier diagnosis may be possible, and how this might be achieved.

## INTRODUCTION

Delayed diagnosis has been associated with poorer prognosis and patient experience in different conditions, such as psoriatic arthritis, Guillain-Barré syndrome, and coeliac disease,^1^^–^3 making strategies to improve diagnosis an important research priority. A diagnostic window is a period during which there is a detectable increase in healthcare use before the diagnosis of an underlying condition in a population of (as-yet-undiagnosed) patients. A diagnostic window indicates the point at which diagnosis may be theoretically possible in at least some patients with the condition^4^ and can help identify conditions or patient groups that may benefit from targeted interventions to achieve earlier diagnosis.

The concept of diagnostic windows is applicable to any disease; however, most existing evidence relates to cancer.^4^^–^7 Diagnostic windows typically range in length from a few months to 3–4 years.^8^ Diagnostic windows can be defined by any type of healthcare-use event (for example, primary care visits, symptoms, abnormal test results), with the type of event determining how the window should be interpreted. For example, when all types of healthcare encounters are considered, the diagnostic window indicates when the as-yet-undiagnosed population starts to use health care in a different way from a baseline healthy population, indicating when at least some as-yet-undiagnosed patients start to seek help and interact with the healthcare system because of their underlying condition. In contrast, for more specific healthcare events, such as ordering a test or a consultation for certain symptoms, the diagnostic window indicates when the as-yet-undiagnosed population undergo the specific test or present with that symptom at higher rates than the baseline healthy population.

By identifying published studies that contained any examination of prediagnostic healthcare utilisation, the aim of the study was to extract evidence on the presence and length of diagnostic windows in non-neoplastic conditions. A secondary aim was to describe the types of healthcare events used to define diagnostic windows.

## METHOD

A systematic review was conducted according to the Preferred Reporting Items for Systematic Reviews and Meta-Analyses (PRISMA) checklist.^9^ The objective of the literature search was to identify research studies that contained evidence of healthcare utilisation for patients over a period immediately before diagnosis of a non-neoplastic condition.

**Table:** How this fits in

Improving timeliness of diagnosis is imperative across disease types. This review identified that for a range of non-neoplastic conditions healthcare use starts to increase in the time before diagnosis. For some conditions, this increase may first start to occur many years before diagnosis. Further research is needed to produce accurate estimates of how much earlier diagnosis may be possible.

### Concept mapping and search term selection

In a preliminary phase, appropriate search terms were developed using concept mapping guided by a previous systematic review on diagnostic windows for cancer^8^ and four exemplar studies, summarised in Box 1.^10^^–^13 The selected exemplar studies all present evidence on the occurrence of defined healthcare events, such as recorded symptoms or healthcare encounters, during specified periods before the diagnosis of a given condition (or before the relevant index date for controls). The reported results all allow for an examination of whether healthcare utilisation changed before diagnosis.

**Box 1.** Summary of exemplar studies

**Study**	**Condition**	**Aims**
**Schrag *et al* (2015)^10^**	Parkinson’s disease	*‘Assess the association between several prediagnostic features and a subsequent diagnosis of Parkinson’s disease, and chart the timeline of these presentations before diagnosis.’*
**Blackwell *et al* (2021)^11^**	Inflammatory bowel disease (IBD)	*‘Examine the prevalence of gastrointestinal symptoms attributable to undiagnosed IBD before diagnosis and … identify predictors for timely specialist review.’*
**Miller *et al* (2021)^12^**	Tuberculosis	*‘Propose a population-based approach for estimating the incidence and duration of diagnostic delays associated with tuberculosis, and … describe the risk factors associated with patients experiencing a diagnostic delay.’*
**Miller *et al* (2021)^13^**	Herpes simplex encephalitis (HSE)	*‘Determine the incidence of diagnostic delays for HSE … identify risk factors and outcomes associated with diagnostic delays.’*

PubMed was searched (25 March 2022) from inception; the final search term (Supplementary Box S1) consisted of four components. The first focused the search on studies that contained evidence from the most relevant areas of health care to this review: primary health care and internal medicine. This was because of the authors’ hypothesis that patients are likely to have most initial interactions within these specialties. The second selected studies that were likely to contain evidence of healthcare utilisation, and the third removed studies relating to conditions outside the scope of this review. A fourth component filtered out irrelevant study designs, such as case reports and randomised controlled trials.

### Study selection

Titles and abstracts were screened to remove papers that did not contain evidence of healthcare utilisation. For example, studies of proposed new tests or algorithms and evaluations of educational interventions to improve diagnosis were excluded. Subsequently, the full texts of remaining papers were assessed for eligibility (Supplementary Table S1). Initially 100 randomly selected titles were screened to develop consensus rules for exclusion and inclusion. The remaining titles, abstracts, and full articles were then screened, with any uncertain cases resolved by discussion with a second reviewer.

### Other study identification methods

Finally, a graph of similar papers was produced for each included study, using the Connected Papers website (17 May 2022)^14^ — an automated approach to citation tracking. The titles, abstracts, and full texts of all yielded ‘connected papers’ were screened to identify other relevant studies, following the same procedure described above.

### Data extraction and synthesis

Details of the study design, aims, setting, sample size, type of prediagnostic healthcare events examined, and the methods used to measure and evaluate prediagnostic healthcare utilisation were extracted from each study. Risk of bias was assessed by a single reviewer using the JBI critical appraisal checklist for case-control studies.^15^

**Figure 1.**
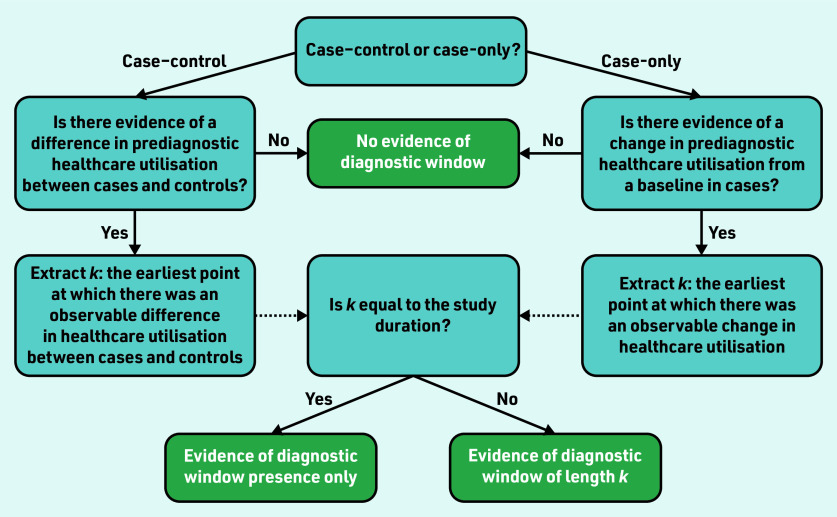
*Flow diagram for determining diagnostic window presence and length.*

Evidence of a difference or change in prediagnostic healthcare utilisation in cases from a measured baseline was used to determine the presence of a diagnostic window. Baseline healthcare utilisation could be measured in controls in a case–control study, or from historic healthcare use in cases in a case-only study. The earliest point relative to diagnosis at which a difference or change from the measured baseline was observed and sustained was extracted. Figure 1 shows how this was used to determine diagnostic window presence and length.

Where estimates of the timing of differences or changes reported in reviewed studies differed between the text and figures or tables of the same publication, evidence reported in the main text was prioritised. Where studies reported evidence on types of healthcare-use events or in different patient groups, evidence of all possible diagnostic windows is reported in the current review.

A narrative synthesis described results across studies, and estimates of diagnostic window length were visualised. As diagnostic windows will differ across conditions, healthcare systems, and the different types of healthcare-use event considered, numerical synthesis was not deemed appropriate.

## RESULTS

**Figure 2.**
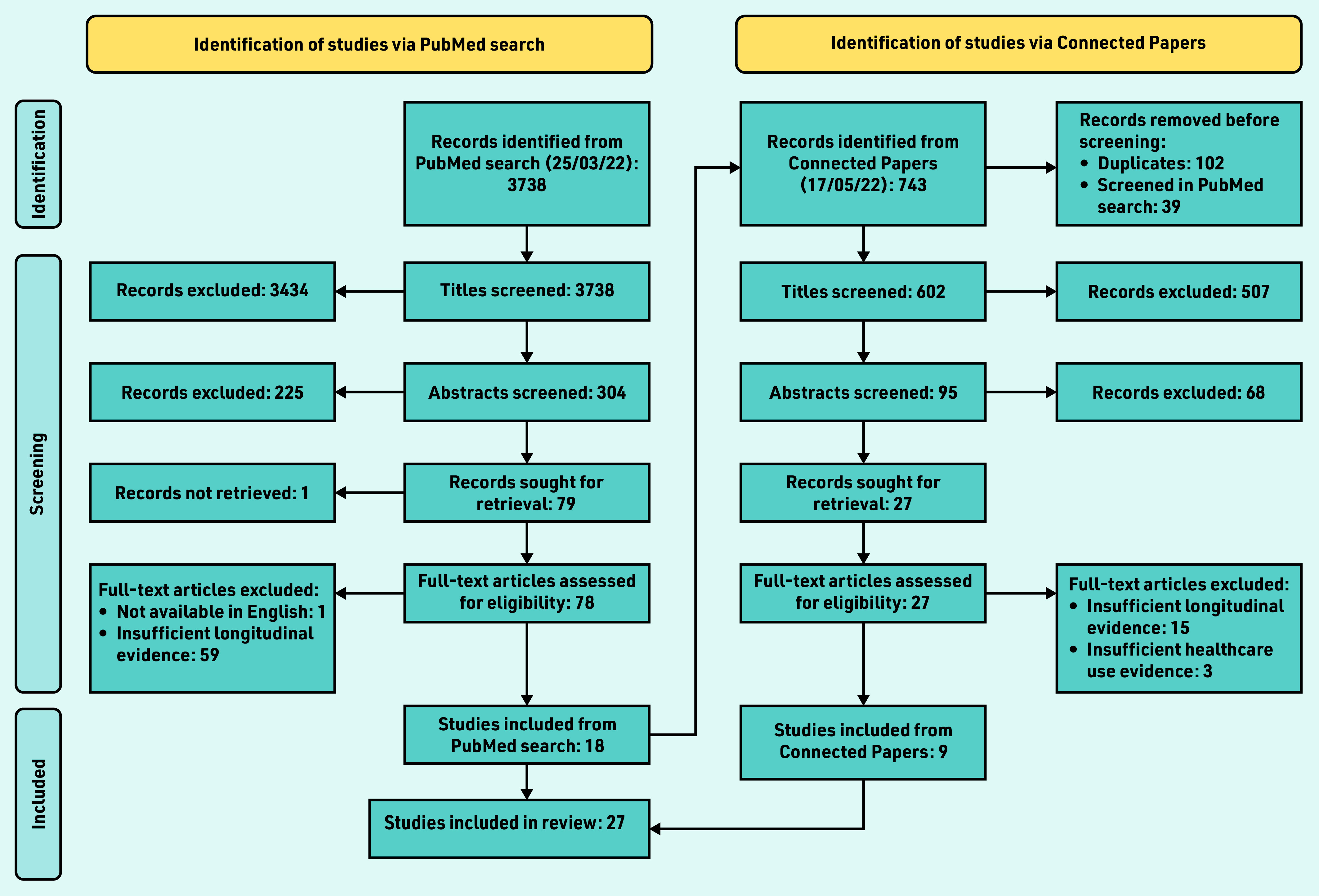
*PRISMA flowchart for literature review of diagnostic windows in non-neoplastic conditions.* *9*

A PRISMA diagram outlines the study search and inclusion process (Figure 2). The PubMed search identified 3738 papers; 3659 papers were excluded in title and abstract screening. Of the remaining 79 papers, 61 were excluded in full-text screening — almost exclusively because they considered the entire prediagnostic time period *en bloc*. By considering the ‘connected papers’ of the 18 included papers (four of which are the exemplar papers noted previously) a further nine papers were identified for inclusion.

Selected studies were published between 2007 and 2022. Total sample size ranged from 568 patients (319 cases)^16^ to 459 774 patients (21 894 cases)^17^ in case–control studies, and from 2667 cases^13^ to 367 768 cases^18^ for case-only studies; sample sizes varied with data source and disease incidence. Most of the included studies were of moderate quality (Supplementary Table S2). The most common issue was the lack of identification of confounding factors or strategies to deal with them (*n* = 16).

### Countries and data sources

Of the 27 included studies, 13 were conducted in the UK,^10^^,^^11^^,^^16^^,^^19^^–^28 of which all but one used longitudinal primary care electronic health record datasets, such as the Clinical Practice Research Datalink and The Health Improvement Network database. In Europe, three studies conducted in Denmark used national registries^17^^,^^29^^,^^30^ and one in Germany used statutory insurance claims data.^31^ Six studies conducted in Canada used centralised administrative and clinical databases of hospital visits, physician billing claims, and prescriptions, with data availability determined at province level.^32^^–^37 Three papers from the US and one paper from Singapore used insurance claims data.^12^^,^^13^^,^^18^^,^^38^

### Conditions

Studies measured prediagnostic healthcare utilisation for 17 different conditions: acute myocardial infarction, childhood type 1 diabetes mellitus, chronic obstructive pulmonary disease, clinically isolated syndrome, coeliac disease, common variable immunodeficiency, dementia, granulomatosis with polyangiitis, herpes simplex encephalitis, inflammatory bowel disease, multiple sclerosis, Parkinson’s disease, psychosis, rheumatoid arthritis, schizophrenia, stroke, and tuberculosis.

### Measures of healthcare utilisation

The main type of healthcare-use event considered was healthcare encounters, although several subtypes were noted: all-cause healthcare encounters versus encounters for specific symptoms, signs, comorbidities, diagnoses, or International Classification of Diseases-10 chapter; encounters in primary care, secondary care, or specific clinical specialties; and encounters via any medium versus face-to-face only. A small number of studies considered other aspects of healthcare use, such as healthcare costs or prescriptions dispensed. Half of the studies considered multiple types of healthcare-use events, for example, daytime GP contacts, out-of-hours GP contacts, and secondary care contacts;^17^ or primary care contacts with individual symptoms.^10^^,^^23^

Several papers estimated rates or rate ratios for healthcare-use events, typically using either Poisson or negative binomial regression. Others estimated the prevalence of healthcare-use events in different time intervals (typically by year pre-diagnosis). Three studies fitted logistic regression models to measure the odds of having a healthcare-use event in specific time intervals in cases compared with controls. Few papers explicitly attempted estimation of diagnostic window length, with the exception of the series of papers from Miller *et al* using change-point detection methods.^12^^,^^13^^,^^18^

### Diagnostic windows

All included studies contained evidence of a sustained difference in prediagnostic healthcare utilisation compared with a baseline ‘healthy’ utilisation for at least one type of healthcare-use event. A summary of study results is provided in Supplementary Table S3. For 15 studies this difference existed for the entire lookback period (study duration), ranging in length from 1 to 10 years, meaning diagnostic windows for these conditions may be longer than their respective study duration(s) and it was not possible to estimate the full length of the diagnostic window (Figure 1). Card *et al* observed an excess of new diagnoses of irritable bowel syndrome in patients subsequently diagnosed with coeliac disease compared with controls from the start of the 10-year study duration.^27^ For two additional studies, ranges were identified that likely contained inflection points, for example, there was evidence that balance problems first become detectable 5–10 years before the diagnosis of Parkinson’s disease.^10^

In the remaining 10 studies at least one diagnostic window was identified. In five studies, a diagnostic window was identified for at least one type of healthcare-use event or patient group, but for other healthcare events or patient groups differences extended for the entire study duration and it was not possible to make any further conclusions on diagnostic window length. For example, Yusuf *et al* showed a change in physician visits for anaemia 2 years before diagnosis of multiple sclerosis and that differences in physician visit rates for fatigue, sleep disorders, and pain existed for 5 years before diagnosis; however, the study duration was 5 years.^33^

**Figure 3.**
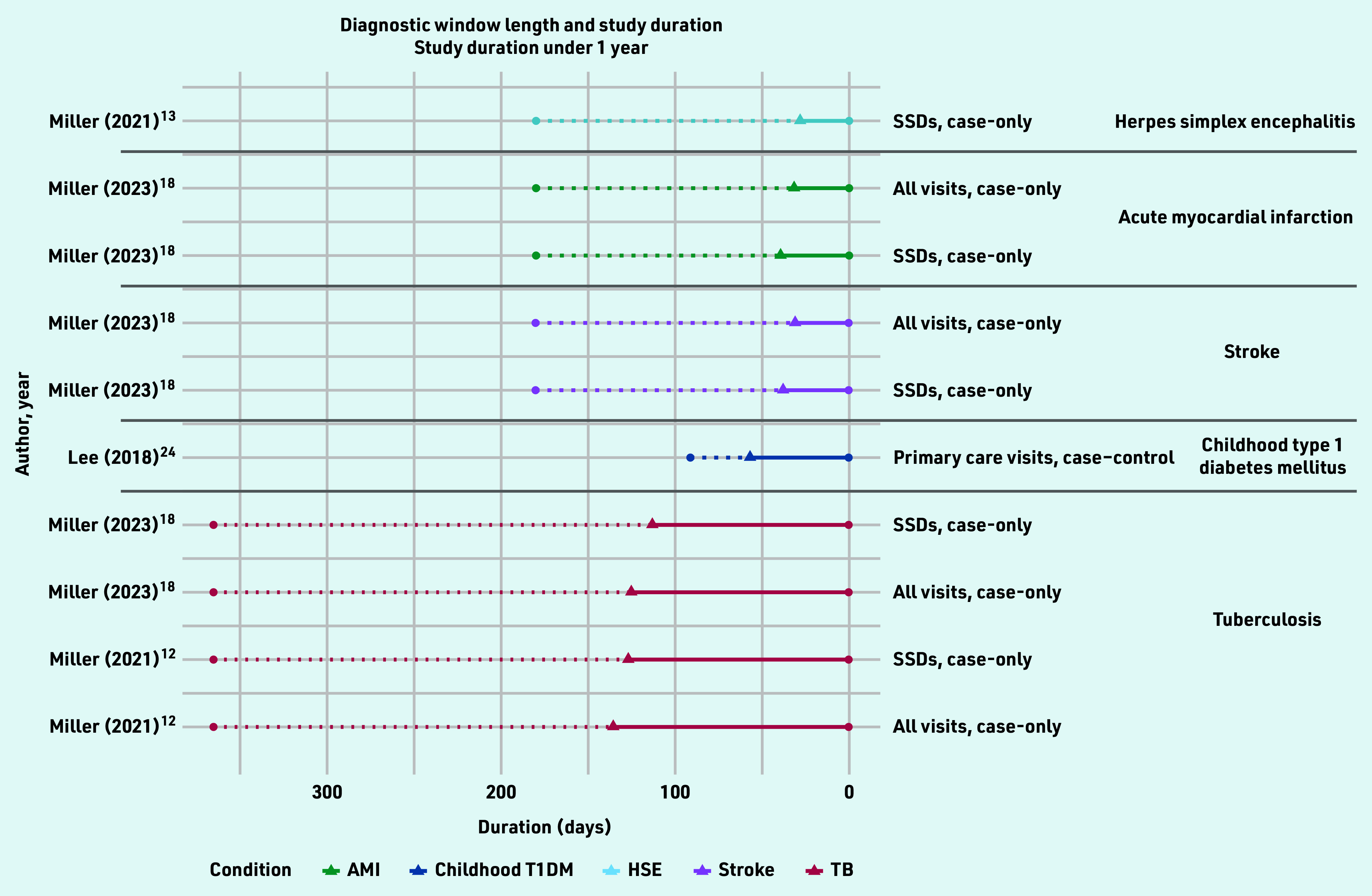
*Identified diagnostic windows and corresponding study duration (under 1 year). Solid lines indicate a diagnostic window; dotted lines indicate the total study duration. The type of healthcare-use event and study design used are given on the right, with different colours denoting different conditions. AMI = acute myocardial infarction. HSE = herpes simplex encephalitis. SSD = symptomatically similar diagnoses.12* *TB = tuberculosis. T1DM = childhood diabetes mellitus type 1.*

**Figure 4.**
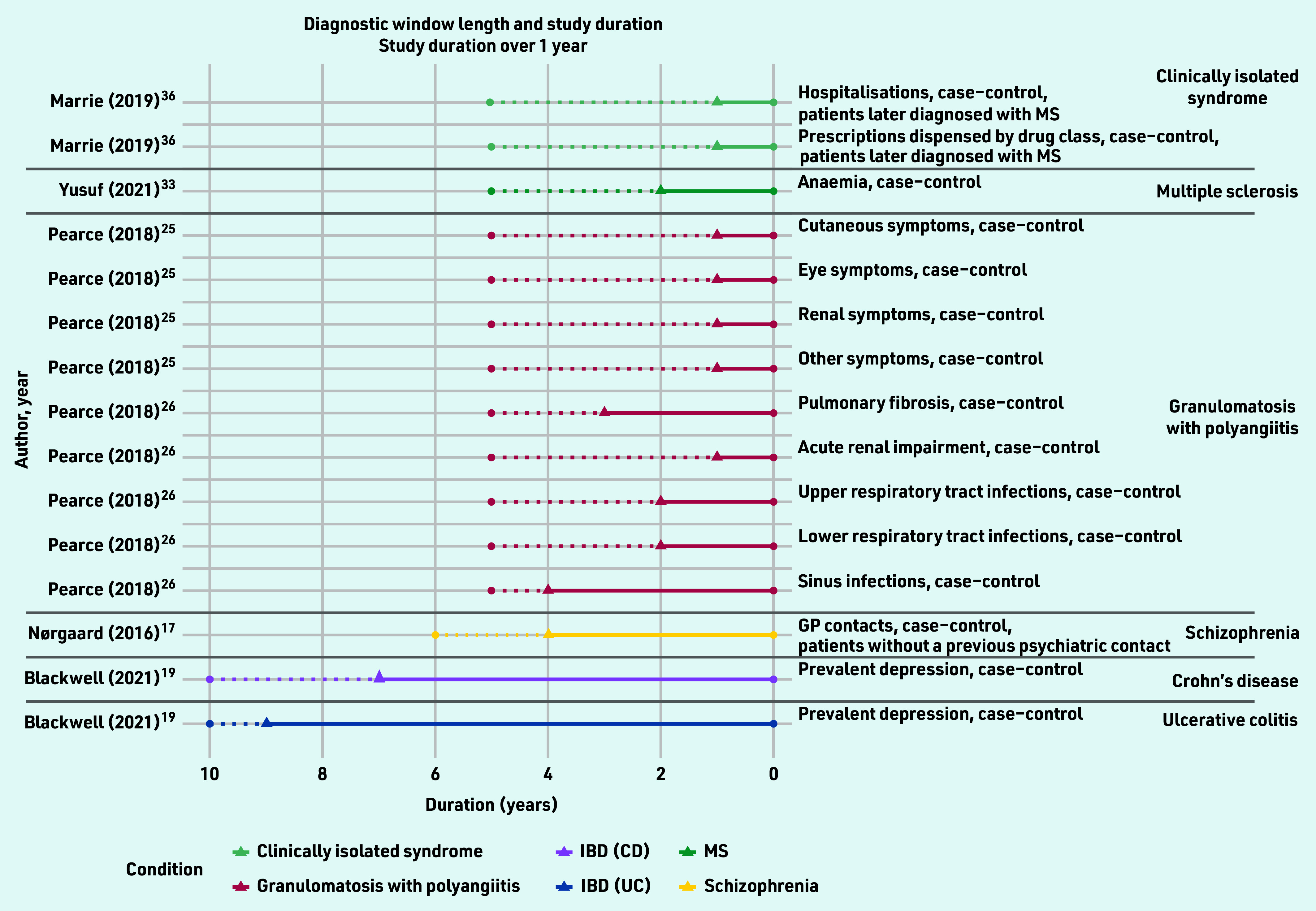
***Identified diagnostic windows and the corresponding study duration (over 1 year). Solid lines indicate a diagnostic window; dotted lines indicate the total study duration. The type of healthcare-use event and study design used are given on the right, with different colours denoting different conditions. CD = Crohn’s disease. IBD = inflammatory bowel disease. MS = multiple sclerosis. UC = ulcerative colitis***.

For the remaining five studies inflection points were identified. In total, 25 diagnostic windows were identified for 10 different conditions over 10 studies, ranging in length from 28 days to 9 years (Figure 3 and Figure 4, Supplementary Table S3).

## DISCUSSION

### Summary

Evidence from health records indicates that diagnostic windows are likely to exist for other conditions and are not unique to cancer.

For acute conditions these windows are likely to be relatively short (<1 year),^12^^,^^13^^,^^18^ but for chronic conditions — in particular autoimmune conditions — windows may span several years.^19^^,^^33^

Some conditions, such as Parkinson's disease and coeliac disease, may be detectable in some patients many years earlier than they are currently diagnosed.^10^^,^^27^ Further research is needed to produce exact estimates of diagnostic windows for non-neoplastic conditions.

### Comparison with existing literature

Literature explicitly examining diagnostic windows for non-neoplastic conditions is limited, and the authors of the current study are not aware of any previous reviews of this evidence. However, some relevant research has been published since this literature search was carried out, including some formal analyses of diagnostic windows for non-neoplastic conditions such as rheumatoid arthritis and necrotising soft-tissue infections of the genitalia.^39^^,^^40^ Some of these studies found different diagnostic windows for conditions also included in this review. For example, Bohlken *et al*^41^ found slightly longer diagnostic windows for Parkinson’s disease for many symptoms included in Schrag *et al*.^10^ This could be because of differences in healthcare settings, data recording practices, or sample size.

A previous review of diagnostic windows for neoplastic conditions found changes in healthcare use at least 6 months before diagnosis for many common cancer sites, with longer windows present for colorectal cancer, brain tumours, and multiple myeloma.^8^ Considered alongside the current review, this supports the hypothesis that the concept of diagnostic windows is applicable to all disease types.

### Strengths and limitations

To the authors’ knowledge, this is the first evaluation of evidence on the presence and length of diagnostic windows outside cancer. The presented evidence of prediagnostic healthcare utilisation from 27 studies spanning 17 different conditions shows that diagnostic windows likely exist and that some may be several years long.

As the concept of ‘diagnostic windows’ has not been widely considered outside cancer, most studies (*n* = 24) did not use statistical methods to formally estimate diagnostic window length, instead relying on visual identification, which may be suboptimal.^42^ A JBI critical appraisal tool was used to assess study quality. Some studies did not report sufficient statistics for the current review's purpose, and it was therefore necessary to rely on text narrative information or exhibits, which sometimes lacked detail.^26^^,^^29^

Constructing a search term that captured all available evidence of prediagnostic healthcare use, without including large numbers of false positives, was challenging. The search focused on studies with a primary care setting — where patients typically first present — but only one database (PubMed) was searched, and most studies were excluded through title screening. Some additional studies that were not captured in this search were identified from Connected Papers; however, it is likely that some relevant papers were not captured by either of these approaches. In future, the identification of relevant papers could be facilitated by wider use of the term 'diagnostic window', where appropriate, and by expanding searches to cover multiple databases. A further limitation is that risk of bias was only assessed by a single reviewer. As such, risk of bias assessment was not used as an exclusion criterion for study selection and is only provided as additional context for the reader.

Studies considered a wide range of healthcare-use events that can provide useful insights in combination. By first considering all-cause healthcare utilisation (in primary and/or secondary care) the earliest timepoint before diagnosis when the condition becomes detectable in some patients can be estimated. Then considering more specific types of healthcare utilisation related to the condition can elucidate how and when the condition may manifest in different ways. However, because of the large variety of healthcare systems, data sources, phenotypes, and definitions of healthcare-use events, comparing individual diagnostic window estimates between studies would be generally inappropriate.

Finally, most studies evaluated healthcare utilisation at yearly intervals. When the study duration was relatively long (5–10 years), this was sufficient. However, for studies with shorter durations (≤3 years) a difference between cases and controls was sometimes evident, but it was not always possible to discern when a change first occurred.^29^^,^^38^ Additionally, most studies (*n* = 20) did not justify their chosen study duration.

### Implications for research and practice

Estimating diagnostic windows can help identify conditions for which future diagnostic quality and safety research efforts should be pursued. However, it should not be assumed that a condition with a longer diagnostic window necessarily represents a more promising target for diagnostic improvement. Every condition has a unique diagnostic process and, when identifying research priorities, the diagnostic window length should be considered alongside the potential consequences of a delayed diagnosis (disease progression, treatment options, and prognosis) and the nature of the condition (chronic versus acute, rare versus common).

Questions remain as to how diagnostic windows of different lengths should be interpreted. As noted in White *et al*, diagnostic window length can vary by patient factors (age, sex, socioeconomic status, comorbidities), healthcare factors (location, size, and type of healthcare setting), disease factors (subtypes, typical disease course), and study era.^8^ Further research is needed to, first, produce accurate estimates of diagnostic window presence and length for non-neoplastic conditions, and, second, examine how these windows are associated with relevant factors, to help identify targets for diagnostic improvement efforts.

Although the existence of diagnostic windows for a given condition does not prove that any individual patient could have been diagnosed earlier, it provides ‘proof-of-concept’ that earlier diagnosis is possible for some patients, while pointing to possible avenues for improvement. Further research is required to identify missed diagnostic opportunities for specific conditions.

Evidence exists of changing healthcare utilisation before the diagnosis of several non-neoplastic conditions — including acute, autoimmune, and mental health conditions — documenting the potential for earlier diagnosis.

The ‘diagnostic windows’ of some of these conditions may span several years and further research is needed to accurately estimate their presence and length. These realisations lay the groundwork for future diagnostic quality and safety research aimed at identifying targets for interventions to improve diagnosis.

## Data

All data included in this review paper are accessible through the literature sources cited.
